# Supplementary material for: Boosting lithium ion conductivity of antiperovskite solid electrolyte by potassium ions substitution for cation clusters
Source: Nat Commun. 2023 Oct 26;14:6807. doi: 10.1038/s41467-023-42385-1 (PMC10603071; doi:10.1038/s41467-023-42385-1)
Supplement: Supplementary file 1 — Supplementary Information [file 41467_2023_42385_MOESM1_ESM.pdf]

Supplementary Information

Boosting lithium ion conductivity of antiperovskite solid electrolyte by potassium ions substitution for cation clusters

Lei Gao, Xinyu Zhang, Jinlong Zhu, Songbai Han*, Hao Zhang, Liping Wang, Ruo Zhao, Song Gao, Shuai Li, Yonggang Wang, Dubin Huang, Yusheng Zhao, Ruqiang Zou*

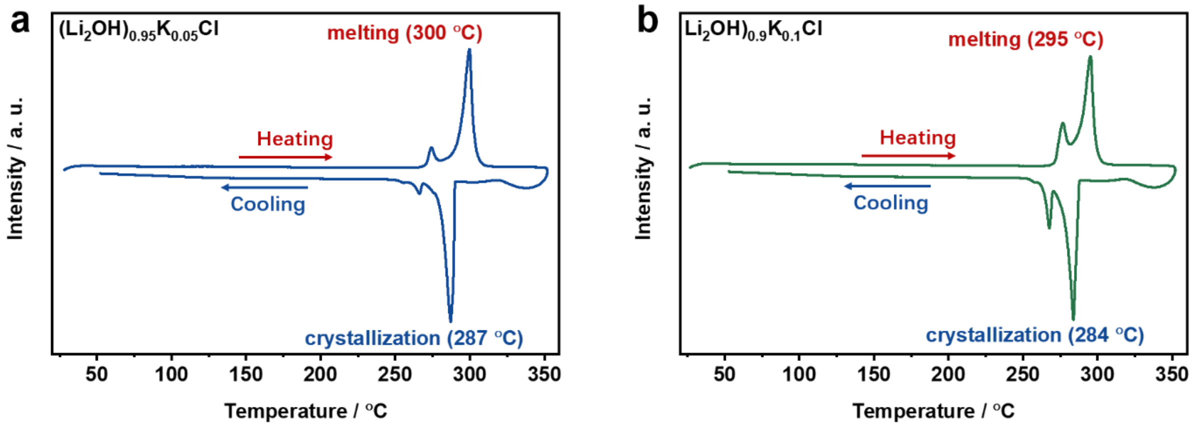

Supplementary Fig. 1 a, b DSC curves of $(Li_2OH)_{0.95}K_{0.05}Cl$ **(a)** and $(Li_2OH)_{0.9}K_{0.1}Cl$ **(b)** between RT and 350 °C.

Supplementary Table 1. Crystallographic information for Li_2OHCl from Rietveld refinement of PXRD.

Total refinement results: R_{wp}:4.06%; R_p:2.18%; GOF:4.78

Li_2OHCl, orthorhombic, $PmC2_1$, a = 3.8719(1) Å, b = 3.8237(1) Å, c = 7.9959(3) Å

Atoms	Wyckoff	*x*	*y*	*z*	Occupancy	U_{iso}
Cl	2b	0.5	0.5	0.25	0.94(1)	0.013(1)
O	2a	0	-0.017(1)	0	1.0	0.009(4)
Li1	2a	0	0.06(2)	0.25	0.75	0.06(2)
Li2	2b	0.5	0.06(4)	0	0.76	0.14(4)

Supplementary Table 2. Crystallographic information for $(Li_2OH)_{0.99}K_{0.01}Cl$ from Rietveld refinement of PXRD.

Total refinement results: R_{wp}:3.98%; R_p:2.49%; GOF:4.15

$(Li_2OH)_{0.99}K_{0.01}Cl$, cubic, *Pm-3m*, *a* = 3.9072(1) Å

Atoms	Wyckoff	*x*	*y*	*z*	Occupancy	U_{iso}
Cl	1b	0.5	0.5	0.5	1	0.026(1)
O	1a	0	0	0	0.99[a]	0.027(4)
Li	3d	0.5	0	0	0.65	0.078(12)
K	1a	0	0	0	0.01[a]	0.027(4)

Supplementary Table 3. Crystallographic information for $(Li_2OH)_{0.95}K_{0.05}Cl$ from Rietveld refinement of PXRD.

Total refinement results: R_{wp}:3.7%; R_p:2.24%; GOF:3.84

$(Li_2OH)_{0.95}K_{0.05}Cl$, cubic, *Pm-3m*, *a* = 3.9036(1) Å

Atoms	Wyckoff	*x*	*y*	*z*	Occupancy	U_{iso}
Cl	1b	0.5	0.5	0.5	1	0.026(1)
O	1a	0	0	0	0.93(3)	0.028(4)
Li	3d	0.5	0	0	0.62	0.059(11)
K	1a	0	0	0	0.066(1)	0.028(4)

Supplementary Table 4. Crystallographic information for $(Li_2OH)_{0.9}K_{0.1}Cl$ from Rietveld refinement of PXRD.

Total refinement results: R_{wp}:3.69%; R_p:2.25%; GOF:2.73

$(Li_2OH)_{0.9}K_{0.1}Cl$, cubic, *Pm-3m*, *a* = 3.8994(1) Å

Atoms	Wyckoff	*x*	*y*	*z*	Occupancy	U_{iso}
Cl	1b	0.5	0.5	0.5	1	0.028(2)
O	1a	0	0	0	0.87(2)	0.027(3)
Li	3d	0.5	0	0	0.57	0.062(9)
K	1a	0	0	0	0.12(1)	0.027(3)

[a]: For $(Li_2OH)_{0.99}K_{0.01}Cl$, because the amount of doped K is low, the occupancy of O and K is set fixed during refinements.

Supplementary Table 5. Crystallographic information for Li_2OHCl from Rietveld refinement of PND.

Total refinement results: R_{wp}:3.17%; R_p:2.10%; GOF:5.09						
Li_2OHCl, orthorhombic, *PmC*2$_1$, *a* = 3.8752(3) Å, *b* = 3.8267(3) Å, *c* = 8.0018(5) Å						
Atoms	Wyckoff	*x*	*y*	*z*	Occupancy	U_{iso}
Cl	2b	0.5	0.5	0.25	0.953(8)	0.0126(6)
O	2a	0	-0.009(3)	0	1	0.009(1)
Li1	2a	0	-0.029(12)	0.25	1	0.042(5)
Li2	2b	0.5	0.077(6)	0	1	0.042(5)
H1	4c	0.161(7)	0	0.068(4)	0.25	0.063(14)
H2	4c	0.161(7)	0	-0.068(4)	0.25	0.063(14)

Supplementary Table 6. Crystallographic information for $(Li_2OH)_{0.99}K_{0.01}Cl$ from Rietveld refinement of PND.

Total refinement results: R_{wp}:2.25%; R_p:1.67%; GOF:2.78						
$(Li_2OH)_{0.99}K_{0.01}Cl$, cubic, *Pm-3m*, *a* = 3.9114(1) Å						
Atoms	Wyckoff	*x*	*y*	*z*	Occupancy	U_{iso}
Cl	1b	0.5	0.5	0.5	1	0.022(1)
O	1a	0	0	0	0.99[a]	0.006(1)
Li	3d	0.5	0	0	0.65(2)	0.093(5)
K	1a	0	0	0	0.01[a]	0.006(2)
H1	6e	0.214(5)	0	0	0.051(6)	0.061(4)
H2	12i	0.149(11)	0.149(11)	0	0.060(1)	0.034(6)

Supplementary Table 7. Crystallographic information for $(Li_2OH)_{0.95}K_{0.05}Cl$ from Rietveld refinement of PND.

Total refinement results: R_{wp}:2.42%; R_p:1.80%; GOF:2.82						
$(Li_2OH)_{0.95}K_{0.05}Cl$, cubic, *Pm-3m*, *a* = 3.9063(1) Å						
Atoms	Wyckoff	*x*	*y*	*z*	Occupancy	U_{iso}
Cl	1b	0.5	0.5	0.5	1	0.024(1)
O	1a	0	0	0	0.941(4)	0.006(1)
Li	3d	0.5	0	0	0.629(18)	0.086(4)
K	1a	0	0	0	0.066(1)	0.006(1)
H1	6e	0.214(5)	0	0	0.051(10)	0.055(12)
H2	12i	0.149(11)	0.149(11)	0	0.058(5)	0.035(6)

Supplementary Table 8. Crystallographic information for $(Li_2OH)_{0.9}K_{0.1}Cl$ from Rietveld refinement of PND.

Total refinement results: R_{wp}:1.97%; R_p:1.46%; GOF:2.84						
$(Li_2OH)_{0.9}K_{0.1}Cl$, cubic, *Pm-3m*, *a* = 3.9023(1) Å						
Atoms	Wyckoff	*x*	*y*	*z*	Occupancy	U_{iso}
Cl	1b	0.5	0.5	0.5	1	0.019(1)
O	1a	0	0	0	0.888(4)	0.006(1)
Li	3d	0.5	0	0	0.594(14)	0.054(3)
K	1a	0	0	0	0.101(7)	0.006(1)
H1	6e	0.214(5)	0	0	0.049(11)	0.052(12)
H2	12i	0.149(11)	0.149(11)	0	0.056(6)	0.032(6)

Supplementary Table 9. Crystallographic information for Li_2OHCl from small-box least square refinement of PDF.

Total refinement results: R_w:14.5%

Li_2OHCl, orthorhombic, *PmC*2$_1$, *a* = 3.896(6) Å, *b* = 3.826(6) Å, *c* = 8.0334(11) Å

Atoms	Wyckoff	*x*	*y*	*z*	Occupancy	U_{iso}
Cl	2b	0.5	0.5	0.25	0.951	0.017(1)
O	2a	0	0	0	1	0.034(3)
Li1	2a	0	0	0.25	1	0.26(12)
Li2	2b	0.5	0	0	0.960	0.04(17)

Supplementary Table 10. Crystallographic information for $(Li_2OH)_{0.99}K_{0.01}Cl$ from small-box least square refinement of PDF.

Total refinement results: R_w:16.0%

$(Li_2OH)_{0.99}K_{0.01}Cl$, cubic, *Pm-3m*, *a* = 3.916(1) Å

Atoms	Wyckoff	*x*	*y*	*z*	Occupancy	U_{iso}
Cl	1b	0.5	0.5	0.5	1	0.022(1)
O	1a	0	0	0	0.99	0.032(2)
Li	3d	0.5	0	0	0.615	0.06(14)
K	1a	0	0	0	0.01	0.032(2)

Supplementary Table 11. Crystallographic information for $(Li_2OH)_{0.95}K_{0.05}Cl$ from small-box least square refinement of PDF.

Total refinement results: R_w:15.7%

$(Li_2OH)_{0.95}K_{0.05}Cl$, cubic, *Pm-3m*, *a* = 3.913(1) Å

Atoms	Wyckoff	*x*	*y*	*z*	Occupancy	U_{iso}
Cl	1b	0.5	0.5	0.5	1	0.022(1)
O	1a	0	0	0	0.942	0.029(2)
Li	3d	0.5	0	0	0.573	0.066(15)
K	1a	0	0	0	0.058	0.029(2)

Supplementary Table 12. Crystallographic information for $(Li_2OH)_{0.9}K_{0.1}Cl$ from small-box least square refinement of PDF.

Total refinement results: R_w:15.2%

$(Li_2OH)_{0.9}K_{0.1}Cl$, cubic, *Pm-3m*, *a* = 3.908(2) Å

Atoms	Wyckoff	*x*	*y*	*z*	Occupancy	U_{iso}
Cl	1b	0.5	0.5	0.5	1	0.023(1)
O	1a	0	0	0	0.892	0.025(2)
Li	3d	0.5	0	0	0.498	0.072(18)
K	1a	0	0	0	0.121	0.025(2)

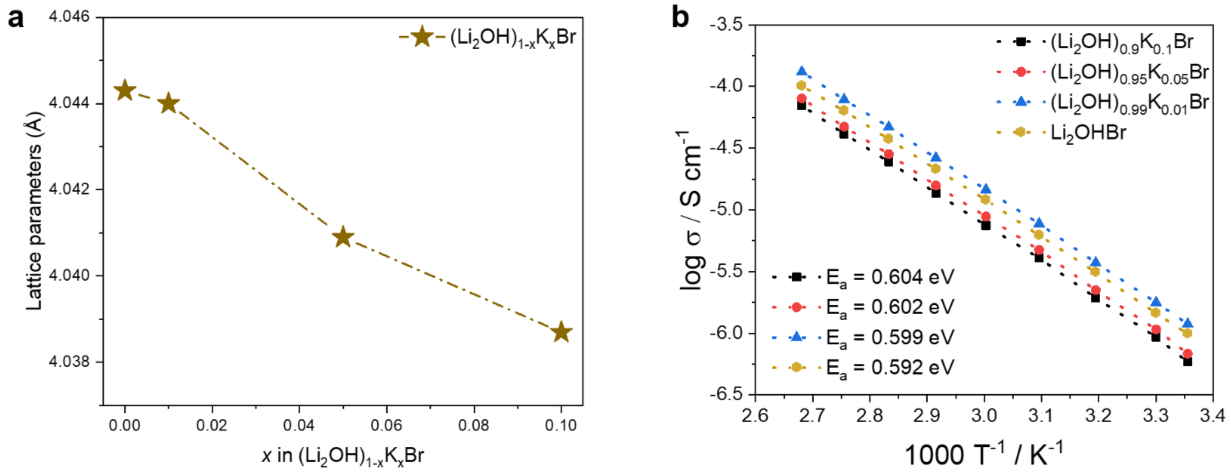

Supplementary Fig. 2 a The lattice parameters of $(Li_2OH)_{1-x}K_xBr$ decreases with the increase of K-doping according to the PXRD. **b** The Arrhenius conductivity plots of $(Li_2OH)_{1-x}K_xBr$ from 25 to 120 °C.

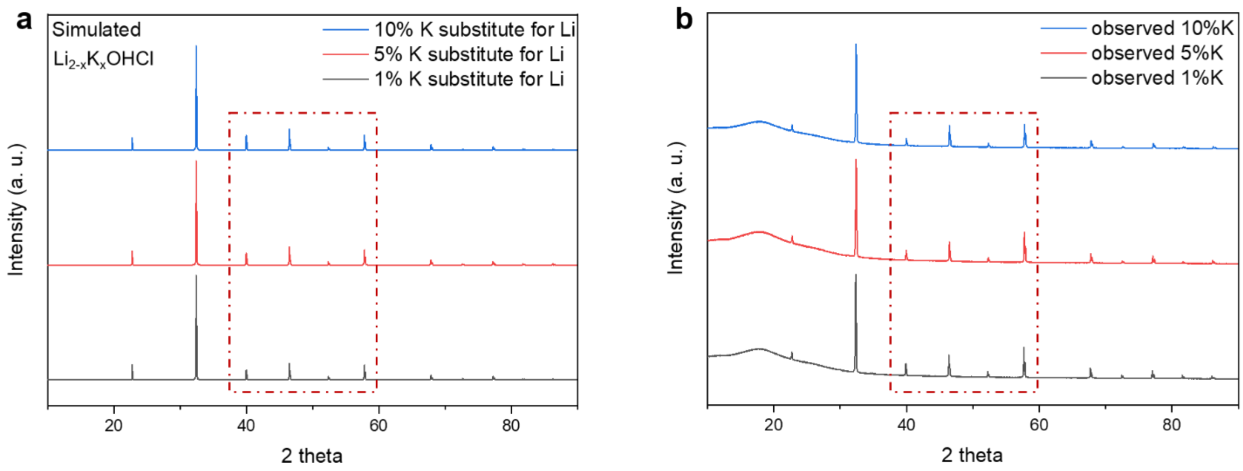

Supplementary Fig. 3 a The simulated PXRD according to the assumed structure model of K substitution for Li sites in antiperovskite lattice. **b** The observed PXRD for comparison. The distinct different characteristics (diffraction peaks from 40° to 60°) between simulated PXRD and observed PXRD negate the hypothesis of K substitution for Li sites in antiperovskite lattice.

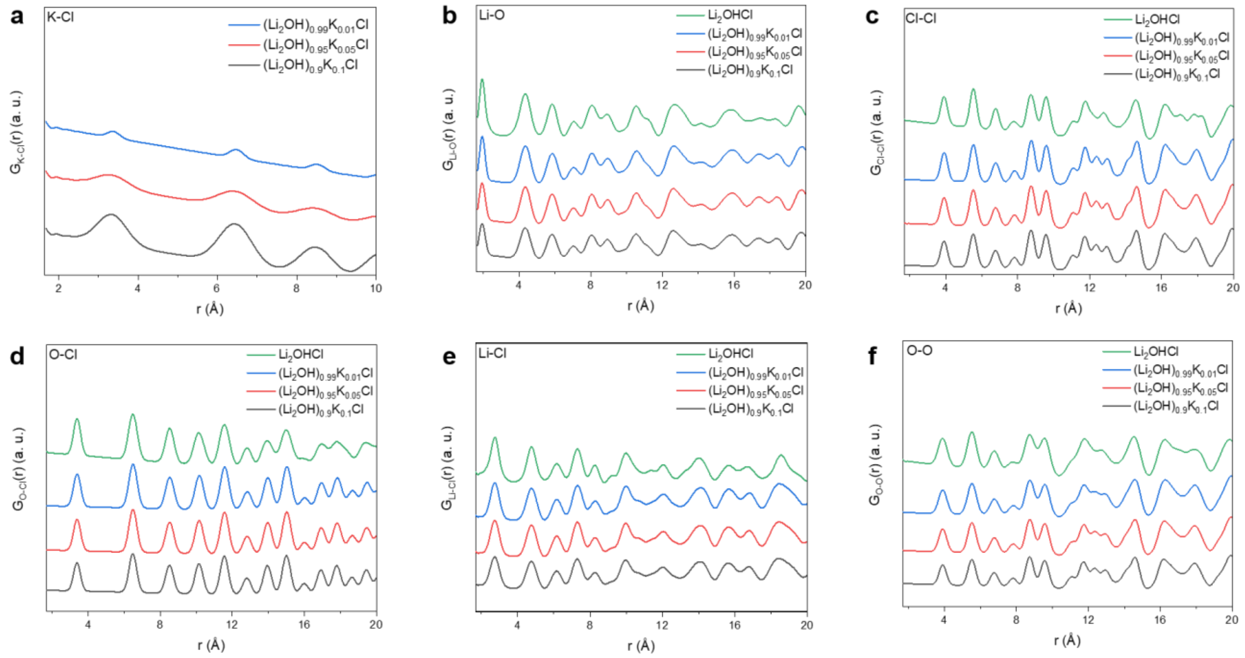

Supplementary Fig. 4 a-f The partial PDF data analysis of K–Cl **(a)**, Li–O **(b)**, Cl–Cl **(c)**, O–Cl **(d)**, Li–Cl **(e)**, and O–O **(f)** in Li_2OHCl and $(Li_2OH)_{1-x}K_xCl$ structure.

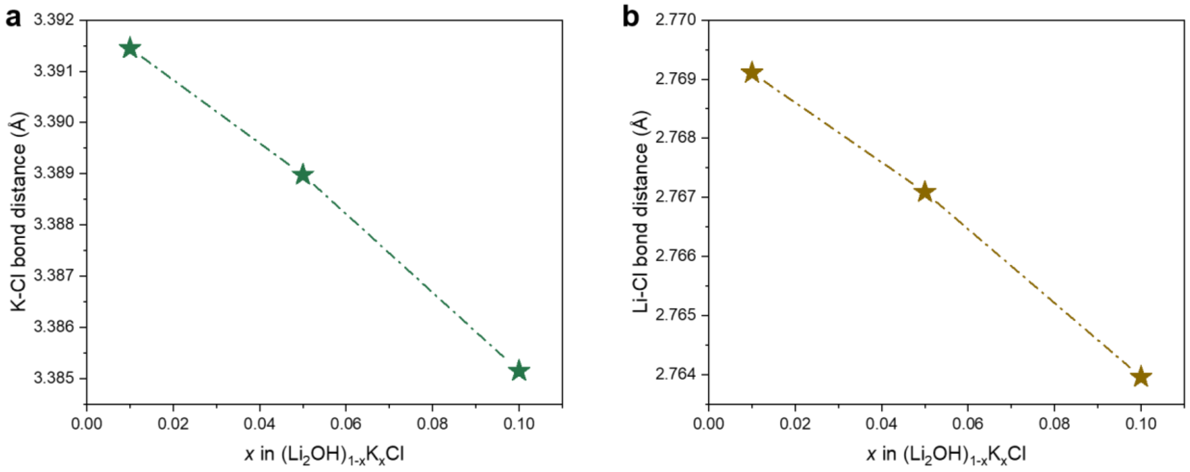

Supplementary Fig. 5 a, b The K–Cl bond **(a)** and Li–Cl bond **(b)** distance according to partial PDF data analysis of $(Li_2OH)_{1-x}K_xCl$.

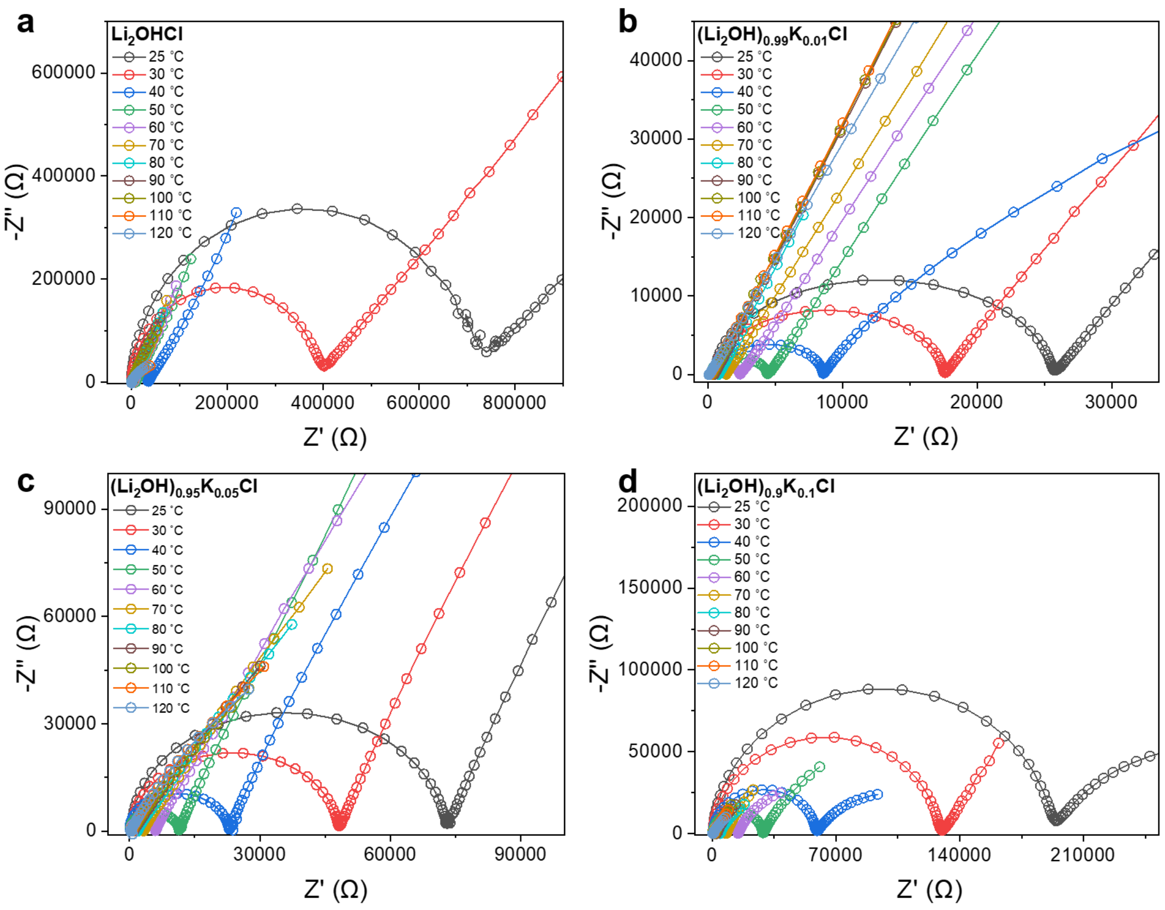

Supplementary Fig. 6 a-d Nyquist plots of Li_2OHCl and $(Li_2OH)_{1-x}K_xCl$ SSEs measured in the temperature range from 25 to 120 °C.

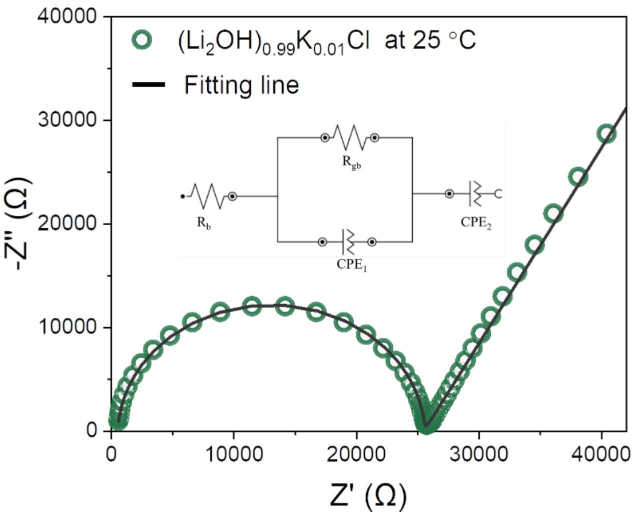

Supplementary Fig. 7 Fitting curve with the equivalent circuit for Nyquist plot of $(Li_2OH)_{0.99}K_{0.01}Cl$ at 25 °C.

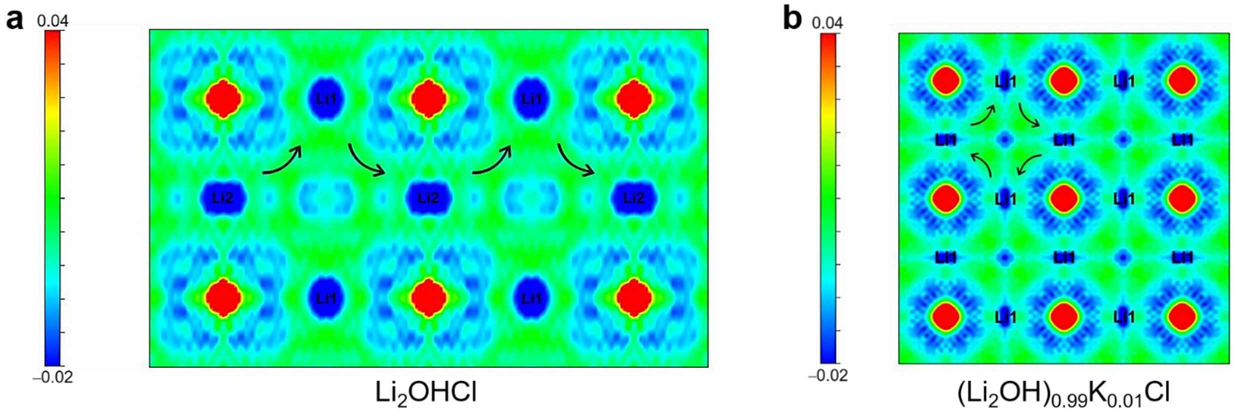

Supplementary Fig. 8 a, b 2D nuclear density maps of Li_2OHCl **(a)** and $(Li_2OH)_{0.99}K_{0.01}Cl$ **(b)** deducted from maximum entropy method analysis. The isosurface level is between –0.02 and 0.04 fm Å^{-3} in a-c plane, and the arrows indicate the preferable Li^+ ions pathways in both structures. The positive density (O atoms) is displayed as red, and the H atoms with negative density (blue) surround the O atoms. The Li atoms with negative density is also displayed as blue, and marked in the figure by Li1 or Li2.

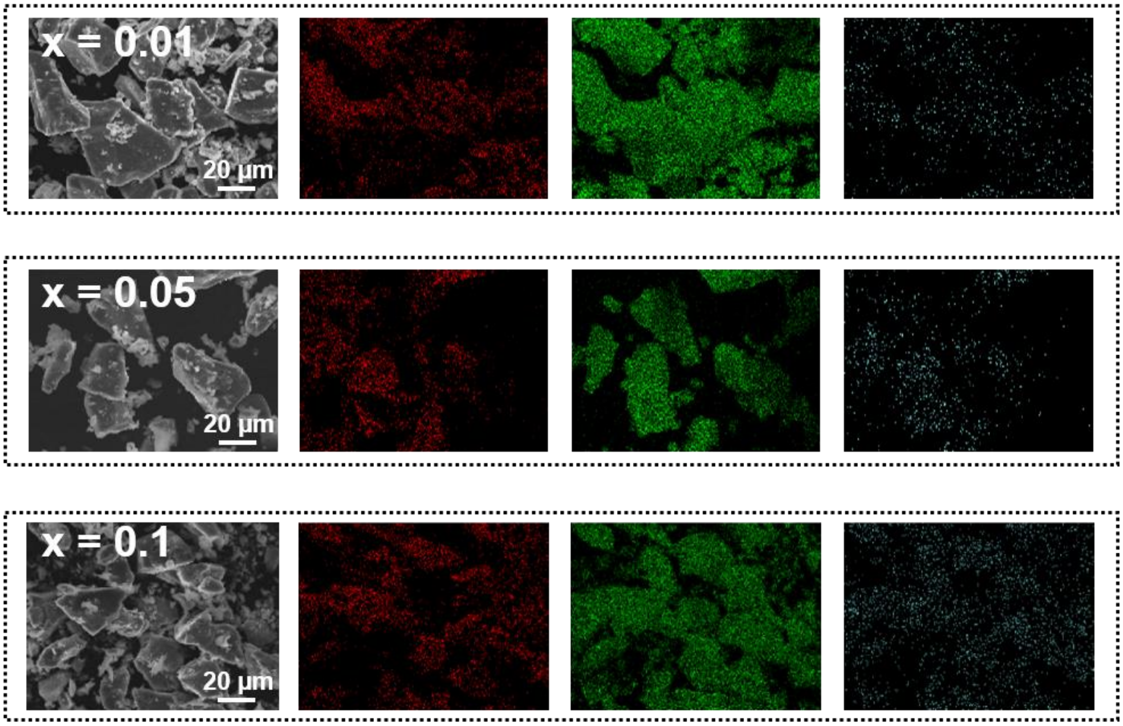

Supplementary Fig. 9 The morphology of $(Li_2OH)_{1-x}K_xCl$ (x = 0.01, 0.05, 0.1) powder observed by SEM, and the energy–dispersive X–ray spectroscopy (EDS) mapping images of oxygen, chlorine, and potassium in red, green, and cyan, respectively.

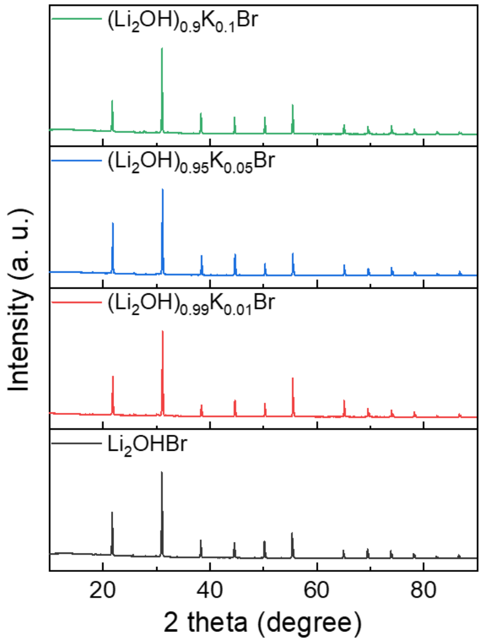

Supplementary Fig. 10 PXRD patterns of cubic Li_2OHBr and $(Li_2OH)_{1-x}K_xBr$ at RT.

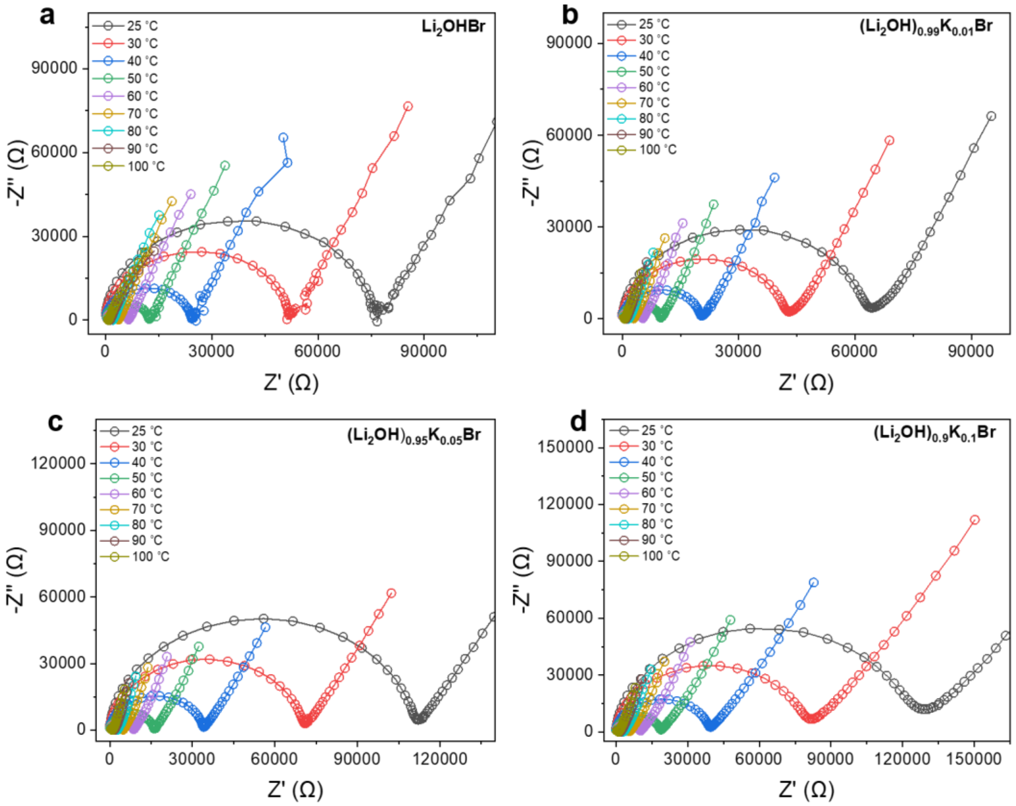

Supplementary Fig. 11 a-d Nyquist plots of Li_2OHBr and $(Li_2OH)_{1-x}K_xBr$ SSEs measured in the temperature range from 25 to 120 °C.

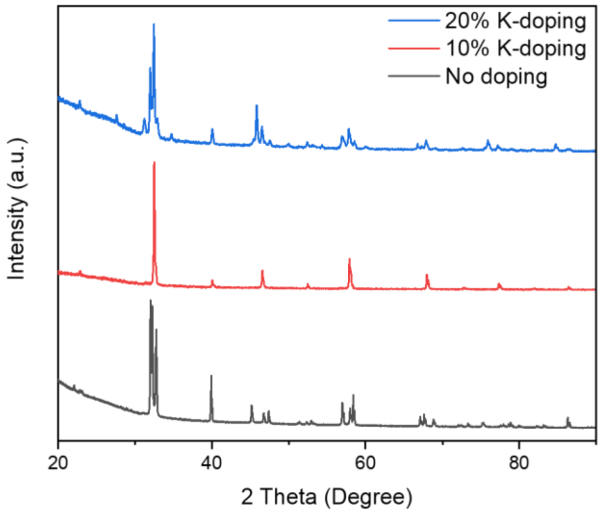

Supplementary Fig. 12 The XRD patterns of Li_2OHCl, 10% K-doping sample and 20% K-doping sample respectively. The XRD results of 20% K-doping sample indicate that the local coordination of K^+ may be unstable after it substitutes the $[Li_2OH]^+$ cluster, when the K-doping concentration is too high.

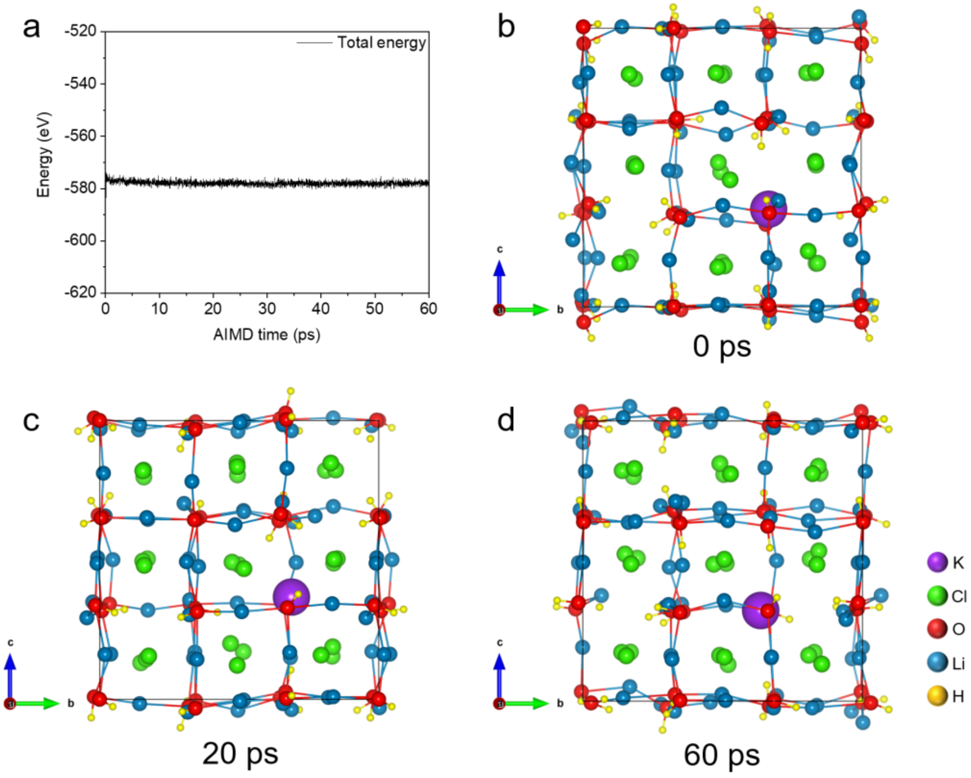

Supplementary Fig. 13 a Evolution of total energy of the proposed K-doped structure at 550 K during the AIMD simulation. **b-d** The extracted structure during the AIMD simulation at 0, 20, and 60 ps.
